# *Gomphrena claussenii*, the first South-American metallophyte species with indicator-like Zn and Cd accumulation and extreme metal tolerance

**DOI:** 10.3389/fpls.2013.00180

**Published:** 2013-06-06

**Authors:** Mina Tomaz Villafort Carvalho, Douglas C. Amaral, Luiz R. G. Guilherme, Mark G. M. Aarts

**Affiliations:** ^1^Laboratory of Genetics, Wageningen UniversityWageningen, Netherlands; ^2^Environmental Geochemistry Laboratory, Soil Science Department, Federal University of LavrasLavras, Brazil

**Keywords:** phytoremediation, Zn/Cd hypertolerance, hyperaccumulation, metal contamination, *Gomphrena claussenii*, *Gomphrena elegans*

## Abstract

Plant species with the capacity to tolerate heavy metals are potentially useful for phytoremediation since they have adapted to survive and reproduce under toxic conditions and to accumulate high metal concentrations. *Gomphrena claussenii* Moq., a South-American species belonging to the Amaranthaceae, is found at a zinc (Zn) mining area in the state of Minas Gerais, Brazil. Through soil and hydroponic experiments, the metal tolerance and accumulation capacities of *G. claussenii* were assessed and the effects on physiological characteristics were compared with a closely related non-tolerant species, *G. elegans* Mart. *G. claussenii* plants grown in soil sampled at the Zn smelting area accumulated up to 5318μgg^-^^1^ of Zn and 287 μg g^-^^1^ of cadmium (Cd) in shoot dry biomass after 30 days of exposure. Plants were grown in hydroponics containing up to 3000 μM of Zn and 100 μM of Cd for *G. claussenii* and 100 μM of Zn and 5 μM of Cd for *G. elegans*. *G. claussenii* proved to be an extremely tolerant species to both Zn and Cd, showing only slight metal toxicity symptoms at the highest treatment levels, without significant decrease in biomass and no effects on root growth, whereas the non-tolerant species *G. elegans* showed significant toxicity effects at the highest exposure levels. Both species accumulated more Zn and Cd in roots than in shoots. In *G. elegans*, over 90% of the Cd remained in the roots, but *G. claussenii* showed a root:shoot concentration ratio of around 2, with shoots reaching 0.93% Zn and 0.13% Cd on dry matter base. In *G. claussenii* shoots, the concentrations of other minerals, such as iron (Fe) and manganese (Mn), were only affected by the highest Zn treatment while in *G. elegans* the Fe and Mn concentrations in shoots decreased drastically at both Zn and Cd treatments. Taking together, these results indicate that *G. claussenii* is a novel metallophyte, extremely tolerant of high Zn and Cd exposure and an interesting species for further phytoremediation studies.

## INTRODUCTION

In many parts of the world, soils have become polluted with high levels of heavy metals mainly due to industrial activities (Ernst, 2006). Most plant species are sensitive to these contaminated conditions whilst certain species have evolved the ability to survive and reproduce in such toxic environments. Such ability can be attained by plants mainly through two strategies: avoidance and tolerance. While species belonging to the first group invest in external mechanisms to keep metals chelated outside, metal tolerant plants developed a physiological machinery adapted to accumulate these high metal concentrations inside the root and/or shoot, dealing with the enhanced stress this will cause (Baker, 1987).

Exposure to high levels of metals is likely to cause alterations in plant physiology. Stunted growth, leaves chlorosis, iron (Fe) deficiency, water unbalance, and reduction of photosynthesis rate are symptoms usually displayed by non-tolerant species when exposed to high levels of zinc (Zn) and cadmium (Cd; Clemens, 2006; Broadley et al., 2007; Gallego et al., 2012). While sensitive species present phytotoxic symptoms with concentrations of Zn from 100 to 400 μg g^-^^1^ and of Cd from 5 to 30 μg g^-^^1^ in shoots, hypertolerant plants can complete their life cycle accumulating more than 3000 μg g^-^^1^ of Zn and/or 100 μg g^-^^1^ of Cd (Kabata-Pendias and Mukherjee, 2007a; van der Ent et al., 2012).

To overcome this stress condition, hypertolerant plants have selected physiological strategies to remove the toxic ions from the most sensitive subcellular parts, such as the cytosol and various organelles (Clemens, 2001). Metal surplus chelation and sequestration into the vacuole or excretion to the apoplast, are mechanisms widely used by hypertolerant species to reduce internal metal bioavailability (Clemens, 2006; Ernst, 2006). Whereas some hypertolerant species accumulate most of the heavy metals inside the root, a particular group, defined as metal hyperaccumulators, have evolved the strategy to translocate and store the metals preferably in the shoot (Brown et al., 1995).

In the last few decades, hyperaccumulator species have received substantial attention because of their interesting metal homeostasis physiology and potential application in phytoremediation, a technology based on the ability of plants to extract or stabilize pollutants in the environment and thus contribute to functional restoration of contaminated areas (Marques et al., 2009).

Phytoextraction theoretically is the ideal remediation technique, capable to reduce soil metal concentrations, at a low cost, to non-toxic levels (Dickinson et al., 2009). To achieve such in an economically viable way, it is crucial to combine traits like high biomass and high metal tolerance and accumulation in the phytoextraction plants (Chaney et al., 2007). At moderately contaminated sites phytoextraction has proved to be feasible using hyperaccumulator species (Rascio and Navari-Izzo, 2010; Hanikenne and Nouet, 2011), however, because such species usually have low biomass production, phytostabilization may be the appropriate technique for severely contaminated soils (Zhao and Mcgrath, 2009). In such a case, plants are used to prevent leaching of pollutants from the soil and provide cover vegetation to improve the soil quality and reduce wind contamination, to further minimize the risk of erosion and leaching leading to contamination of ground and surface waters (Dickinson et al., 2009; Zhao and Mcgrath, 2009).

Hypertolerance has likely evolved independently within different angiosperm families (Ernst, 2006) and often this is a trait present only in one genus or even one species. Some families, such as the Brassicaceae, show a higher occurrence of Zn, Cd, or nickel (Ni) hyperaccumulators species, like the hyperaccumulator models *Noccaea caerulescens* and *Arabidopsis halleri* (Broadley et al., 2001; Assunção, 2003b; Hanikenne and Nouet, 2011).

Researches with hypertolerant and hyperaccumulator species from tropical environments falls far short of what is known about temperate taxa (Baker et al., 2010). Latin America is the least studied continent, with few metallophyte (metal tolerant and/or hyperaccumulator plants) species reported: only 172 species among which 89% are related to Ni. So far no Zn or Cd hyperaccumulator species have been described (Ginocchio and Baker, 2004). Nevertheless, there is no clear geographic reason that Latin America is so poorly represented, as it has a uniquely diverse flora (8 of the 25 biodiversity hotspots in the world are in Latin America) but also due to the presence of countless sites rich in metal ores as well as metal smelter areas (Ginocchio and Baker, 2004; Reeves et al., 2007; Baker et al., 2010).

**FIGURE 1:**
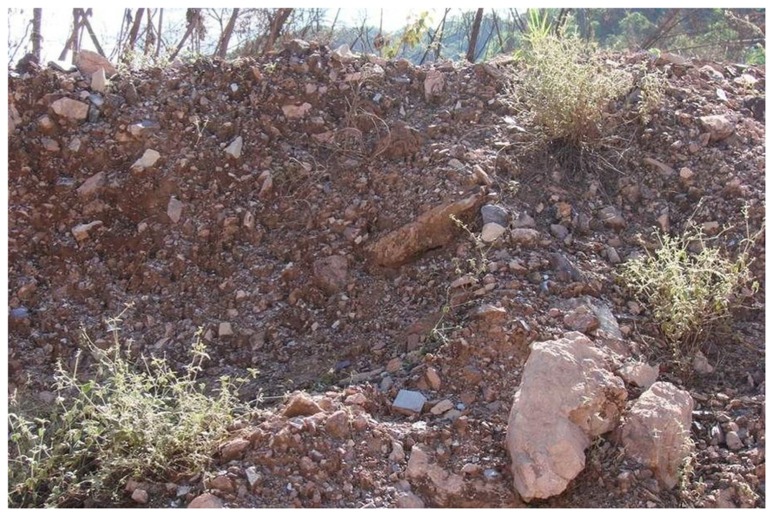
***Gomphrena claussenii Moq. plants growing at a Zn mining site at Vazante, Minas Gerais, Brazil***.

Plants naturally growing in metal-enriched soils are in general metal tolerant, which makes the vegetation native to contaminated areas an important potential source of metal tolerant and accumulator species (Baker and Brooks, 1989). One example is the Zn mining site near Vazante in the state of Minas Gerais (MG), Brazil, where almost all of the Zn extraction in Brazil takes place (Filho and Viana, 2011). One species at this site, *Gomphrena claussenii* Moq. (**Figure 1**) has the ability to grow and thrive at the locally high Zn and Cd levels, making it a potentially interesting species for phytoremediation. *G. claussenii* is a perennial species, belonging to the Amaranthaceae family, and native to Brazil (Marchioretto et al., 2010). *G. elegans* Mart. is a related species, which is widespread in South America, but not reported to be tolerant to excess metal exposure (Mussury et al., 2006). It is used as a metal sensitive species for this study. The work presented here aims to evaluate the physiological effects of high Zn and Cd on *G. claussenii* when compared with *G. elegans*. Consequently, we assess the metal tolerance capacity of *G. claussenii* to toxic metals and evaluate its potential for use in phytoremediation.

## MATERIALS AND METHODS

### PLANT MATERIAL AND GROWTH CONDITIONS

*Gomphrena claussenii* Moq. plants were collected from a Zn mine area at Vazante, in the state of MG, Brazil. *G. elegans* seeds were collected in the field at Antônio João, in the state of Mato Grosso do Sul, Brazil, and provided by Dr. Rosilda Mara Mussury from the Federal University of Grande Dourados, Dourados, Brazil. Seeds could not be used as starting material for *G. claussenii* since field access was limited and seeds are only mature at restricted periods during the year. Instead, seven individual plants were collected at the site and vegetatively propagated. After a pilot experiment and confirmation of the high Zn and Cd tolerance of all plants, one line was brought into *in vitro* tissue culture and taken to The Netherlands for further experiments. *G. elegans* seeds were sterilized and germinated as start material.

Both species were vegetatively propagated through tissue culture using half-strength Murashige and Skoog (MS) medium containing, 2% sucrose and 0.8% agar at pH 5.8. Plant material were cultured in a growth room (24°C, 250 μmol m^-^^2^ s^-^^1^ light at plant level and a 16-h light/8-h dark cycle). Two-week-old tissue culture-grown cuttings were used as starting material for soil and hydroponics experiments.

### Zn AND Cd TOLERANCE

#### Soil Assay

Zinc ore extracted at Vazante is processed in the metal smelter at Três Marias, MG, Brazil. Soil samples were collected at four different sites around the Zn smelter, three contaminated sites and one non-contaminated control site at some distance from the smelter (control: 18°12′16′′S/45°14′02′′W; site 1: 18°11′25′′S/45°14′10′′W; site 2 18°11′08′′S/45°14′07′′W; site 3 18°11′06′′S/45°14′24′′W). Pre-cultured *G. claussenii* plants were planted in the four different soils in 250-ml pots. The experiment was performed with three repetitions, each represented by one pot with one plant, during the winter season in a glass greenhouse, at Lavras Federal University, Lavras, MG, Brazil,. After 30 days, shoots were harvested and washed with demi-water for biomass and mineral concentration measurements.

#### Hydroponic Assay

*Gomphrena claussenii* and *G. elegans* *in vitro* cuttings were transferred to 600-ml polyethylene pots (one plant per pot and three replicate pots per treatment) containing a modified Clark’s full strength nutrient solution (Clark, 1975): 1.3 mM KNO_3_, 2.53 mM Ca (NO_3_)_2_, 0.9 mM NH_4_NO_3_, 0.6 mM MgSO_4_, 0.5 mM KCl, 34.5 μM Ca (H_2_PO_4_)_2_, 19 μM H_3_BO_3_, 2 μM ZnSO_4_, 7 μM MnCl_2_, 0.5 μM CuSO_4_, 0.086 μM (NH_4_)_6_Mo_7_O_24_, and 38μM Fe(Na) ethylenediaminetetraacetic acid (EDTA). The pH buffer 2-(*N*-morpholino)ethanesulfonic acid (MES) was added at 2 mM and the pH was set at 5.5 using potassium hydroxide (KOH). After 3 weeks growing on quarter-strength Clark’s solution, plants were exposed to half-strength solution with normal Zn (2 μM) or excess Zn/Cd: 100, 1000, and 3000 μM of ZnSO_4_ or 10, 50, and 100 μM of CdSO_4_ (at 2 μM ZnSO_4_) for *G. claussenii* and 100 μM of ZnSO_4_ or 5 μM of CdSO_4_ (at 2 μM ZnSO_4_) for *G. elegans*. The applied Zn and Cd concentrations were chosen to be in the range of bioavailable concentrations at the site of collection and based on pilot experiments. The solutions were replaced once a week and plant culture was performed in a climate chamber [20/15°C day/night; 250 μmol m^-^^2^ s^-^^1^ light at plant level; 12 h day length; 70% relative humidity (RH)]. After 3 weeks of metal exposure or control treatment, the plants were harvested. Roots were first desorbed with ice-cold 5 mM PbNO_3_ for 1 h. Solubility of minerals was calculated using the solution speciation software Visual MINTEQ 3.0 (Gustaffson, 2007).

#### Root Elongation

The ability of *G. claussenii* and *G. elegans* to tolerate excess metal exposure was tested through root elongation measurements (Schat and Ten Bookum, 1992). Plants were grown in the same hydroponic conditions as described above, at normal (2 μM of Zn) or the highest metal exposure (3000 μM ZnSO_4_ or 100 μM CdSO_4_/2 μM ZnSO_4_ for *G. claussenii* and 100 μM ZnSO_4_ or 5 μM μM CdSO_4_/2 μM ZnSO_4_ for *G. elegans*). Before metal exposures, roots were stained with active coal powder to allow the measurement of the longest unstained root (Schat and Ten Bookum, 1992). Roots were measured after 3 and 6 days of exposure.

### ASSESSMENT OF MINERAL CONCENTRATIONS

Shoot samples from *G. claussenii* plants were collected for mineral analyses from plants sampled at six different locations at the Zn mining site (Vazante). Plant materials were digested in a CEM^®^ Mars-5 microwave oven system (CEM Corporation, Matthews, NC, USA), following the USEPA 3051 method (USEPA, 1995). For the soil experiment, both plant and soil materials were digested as mentioned before. Metal bioavailability (water-soluble fraction) was estimated from soil solution extracts obtained by the saturated-paste technique (Raij et al., 2001). Filtrates were passed through 0.22-μm cellulose membranes to determine the total dissolved metals. The concentrations of Zn and Cd in all extracts were determined by using either flame or graphite-furnace atomic absorption spectrophotometry (PerkinElmer^®^AAnalyst^TM^800). NIST standard reference materials (SRM 1573a Tomato Leaves, SRM 2710 Montana Soil, SRM 1640 Trace Elements in Natural Water) were used to check the accuracy of elemental determinations, which was found satisfactory, i.e., metal recoveries ranged from 78 to 122%. For the analysis of total metal concentrations in plant samples of the hydroponic experiments, 50–90 mg of each sample was wet-ashed in 2 ml of a 4:1 mixture of HNO_3_ (65%) and HCl (37%), in Teflon bombs for 7 h at 140°C and thereafter had their volume adjusted to 5 ml with demineralized water. Metal concentrations (Zn, Cd, Fe, and Mn) were determined using flame atomic absorption spectrophotometry (PerkinElmer AAnalyst 100; PerkinElmer Nederland, Nieuwerkerk a/d IJssel, The Netherlands).

### STATISTICS ANALYSES

Data was statistically evaluated through analysis of variance (ANOVA) tests following the Tukey’s test used to compare mean values.

## RESULTS

### MINERAL ANALYSES FROM THE FIELD

As expected for metal mining areas there is considerable variation in the soil metal levels at the site and consequently also the plants collected at different locations at the site showed variation for Zn and Cd concentrations in shoots. Plants contained between 230 and 10434 μg g^-^^1^ of Zn and 6 and 96 μg g^-^^1^ of Cd in shoot dry weight samples. Concentrations were correlated with soil levels meaning that higher levels were found in plants growing in more contaminated sites whereas the lowest levels were found in plants collected in an area close to the mine but were mining was not conducted.

### SOIL AND PLANT Zn AND Cd ANALYSIS

Soil samples were taken from the Zn smelter at four different points. Total metal concentrations varied when comparing the different sampling sites and they showed extremely high Zn and Cd levels compared to the control sample (**Table 1**), as was expected for soil sampled at a Zn smelting area. Although slightly higher than metal concentrations normally found in non-contaminated soil, the metal concentrations of the control sample are within the range for non-contaminated soil, even though the sample was taken not far from the industrial area. The highest levels of both metals are found in sample 3, which is taken at the Zn smelter ore waste deposit site, while the other two samples are taken slightly more distant from this site. Comparing to the total levels, the water-soluble (available) metal concentrations were always much lower, except for site 3, at which they were above what is considered to be within the normal range for plants (Kabata-Pendias and Mukherjee, 2007a; **Table 1**). *G. claussenii* plants taken from the site grew well in a greenhouse in pots containing this soil and accumulated up to 5318 μg g^-^^1^ of Zn and 287 μg g^-^^1^ of Cd in their shoots after 30 days of exposure (**Table 1**). Since air contamination was excluded once plants were grown in a greenhouse far away from the Zn smelter, the high metal concentrations can only be caused by high uptake and root to shoot translocation of metals from the soil.

**Table 1:** Zinc (Zn) and cadmium (Cd) concentrations (μg g^–1^) in soil samples from the Zn smelting area at Três Marias, MG, Brazil, and in shoots of *Gomphrena claussenii* plants after growing for 30 days in control and metal contaminated soil collected at four sites around the Zn smelter.

Sample site	Soil (μg g^–1^)				Shoot (μg g^–1^ DW)	
	Total concentration		Available concentration	
	Zn	Cd	Zn	Cd	Zn	Cd
Control	117.7 (2.1)	3.6 (0.4)	0.2 (0.1)	0	22 (1.2)	1 (0.1)
Site 1	3830.1 (62.9)	73 (2.2)	0.6 (0.01)	0.06 (0)	237.9 (1.4)	7.5 (0.1)
Site 2	960.2 (104.3)	12 (1.7)	4.9 (0.1)	0.08 (0)	241.2 (1.1)	7.3 (0.03)
Site 3	15212.9 (580.3)	147.4 (4.4)	168.2 (18.4)	4.63 (0.1)	5318.36 (10.33)	287 (8)

*Mean total and water-soluble (available) soil and shoot metal concentrations are shown, with standard errors between brackets. n = 3. DW, dry weight.*

### Zn AND Cd TOLERANCE IN HYDROPONIC SOLUTION

Exposing plants to hydroponic solutions with high concentrations of metals can be misleading if metals precipitate upon preparing the solution and thus become unavailable to plants. Therefore, we calculated solubility of Zn and Cd in the half-strength Clark’s solution we used as growing medium. Even at the highest Zn and Cd concentrations, both metals were completely soluble and available for uptake (**Table 2**). The main ion forms for those elements in solution are Zn^2^^+^and Cd^2^^+^. These forms are expected to be readily available for plant uptake.

**Table 2:** Zinc (Zn) and cadmium (Cd) speciation in half-strength Clark’s solution containing the highest Zn or Cd concentrations which were used in exposure experiments (3000 μM Zn or 100 μM Cd), as calculated according to Visual MINTEQ 3.0.

Component	% of total concentration	Speciation
Cd^2+^	93.1	Cd^2+^
	1.9	CdCl^+^
	3.7	CdSO_4(aq)_
Zn^2+^	82.4	Zn^2+^
	16.6	ZnSO_4(aq)_

*Speciations with less than 1% were not included. aq, aqueous.*

Root elongation measurements confirmed the strong metal tolerance properties of *G. claussenii*, especially when compared to *G. elegans*. Upon Zn (3000 μM) and Cd (100 μM) treatments *G. claussenii* plants showed no significant effects in root growth (*P* < 0.001; *n* = 6) compared with plants grown under control conditions (**Figure 4**). Instead, roots seemed to grow even longer under high Zn and high Cd. *G. elegans* plants presented a drastic reduction in root growth, already after 3 days of exposure to 100 μM of Zn or 5 μM of Cd (**Figure 4B**). With increased exposure time *G. claussenii* showed no reduction in growth while *G. elegans* plants after 6 days of exposure displayed an even higher reduction in root growth.

**FIGURE 2:**
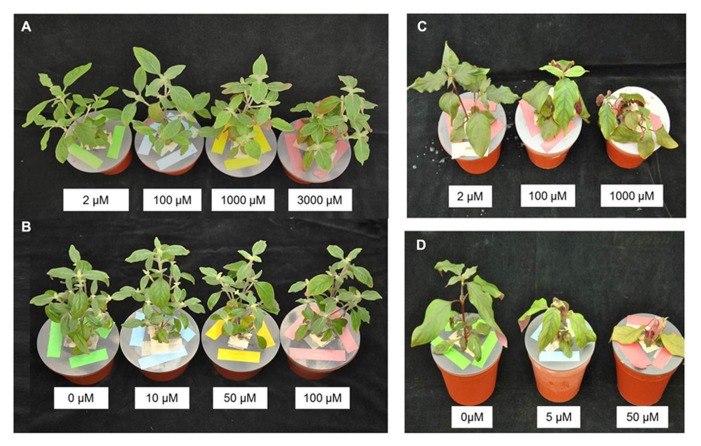
***Gomphrena claussenii* (A,B) and *G. elegans* (C,D) plants after 3 weeks of exposure to different concentrations of zinc (Zn) (A,C) and cadmium (Cd) (B,D).** Plants are grown hydroponically in half-strength Clark’s solution, containing 2 μM Zn, and supplemented to 100, 1000, or 3000 μM Zn, respectively 10, 50, or 100 μM Cd for *G. claussenii* and 100 or 1000 μM of Zn, respectively 5 or 50 μM of Cd for *G. elegans*.

Zn and Cd tolerance was evaluated in *G. claussenii* and *G. elegans* plants based on three parameters: toxicity symptoms, growth rate (dry weight), and root elongation. The *G. claussenii* plants only exhibited slight metal toxicity symptoms, and exclusively at the highest treatment levels (3000 μM Zn or 100 μM Cd; **Figures 2A,B**), confirming their extreme tolerance to both Zn and Cd treatments. *G. elegans* plants already developed visual toxicity symptoms when exposed to 100 μM of Zn and 5 μM of Cd (**Figures 2C,D**). They also showed a reduced growth rate and strong leaves chlorosis, starting in the first week of metal exposure, The toxicity symptoms became more severe with increasing exposure time. In the third week, the oldest leaves started to fall off.

**FIGURE 3:**
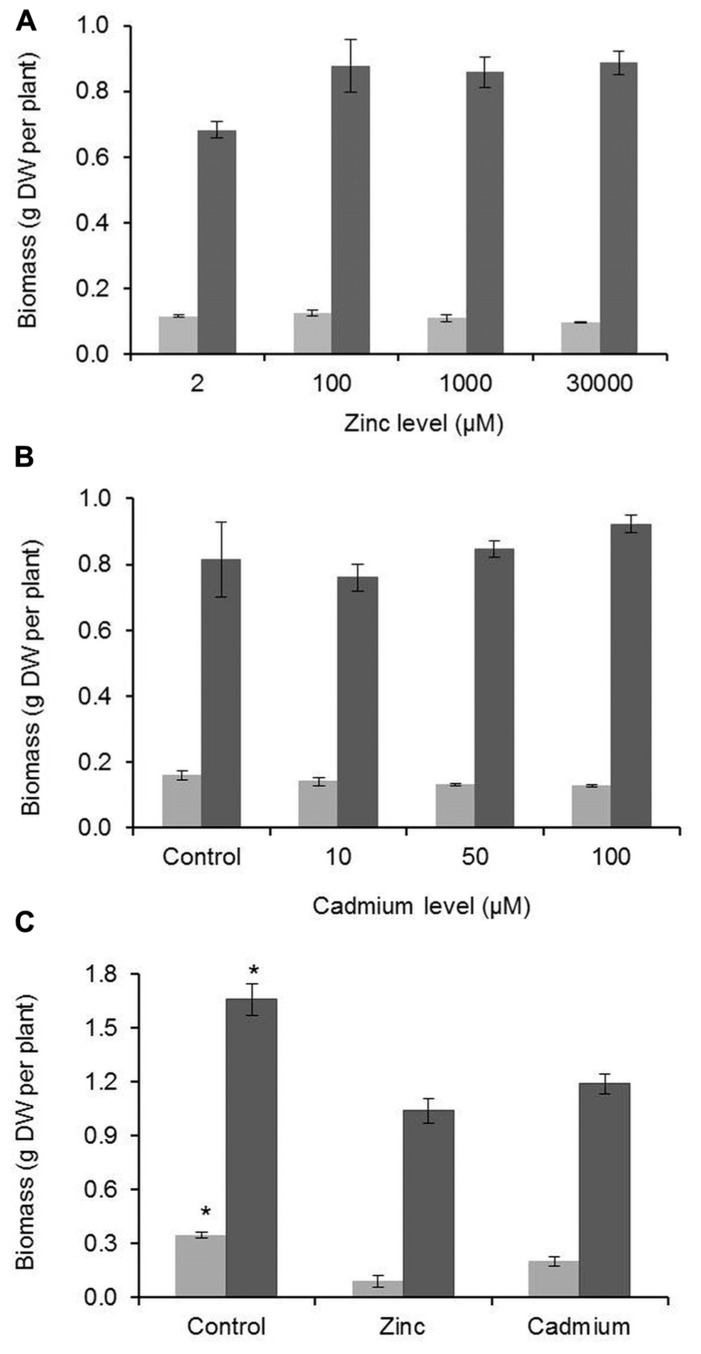
**Root (light gray bars) and shoot (dark gray bars) biomass of *G. claussenii* (A,B) and *G. elegans* (C).** *G. claussenii* plants were exposed to different zinc (Zn; 2–3000 μM) and cadmium (Cd; 0–100 μM) concentrations and *G. elegans* plants were exposed to normal Zn (2 μM), high Zn (100 μM) and Cd (5 μM), for 3 weeks. Data points and error bars represent mean values (*n* = 3) and standard errors, respectively. DW, dry weight. Asterisks denote significant differences of control (2 μM Zn) from treatments as found upon Tukey’s testing (*P* < 0.05).

Growth responses to high Zn and Cd concentrations were different for *G. claussenii* and *G elegans*. As expected for a non-metal-tolerant species, *G. elegans* biomass production decreased notably and significantly (*P* < 0.05; *n* = 3) for shoot and roots when comparing plants grown at high metal exposures with the ones grown in control conditions. Such was not the case for *G. claussenii*, for which an increase in Zn or Cd concentration did not reduce root or shoot dry weight, not even at the highest metals concentrations (**Figure 3**). In fact, the concentration of 2 μM Zn, which is considered to be sufficient for plants in general, may be suboptimal for *G. claussenii* plants, which produce a higher, shoot biomass at elevated Zn concentrations, although the difference was not statistically significant with the low number of plants we tested (*P* > 0.05; *n* = 3).

### MINERAL CONCENTRATIONS

Mineral concentrations were measured from plants growing in hydroponic conditions after 3 weeks of exposure to elevated Zn/Cd conditions. Zn and Cd concentrations increased significantly for both studied species in roots and shoots with increased Zn or Cd exposure levels (**Figure 5**). *G. claussenii* and *G. elegans* showed higher Zn and Cd concentrations in roots than shoots at all treatment levels. For *G. claussenii* the metal concentrations increased nearly proportional with increasing exposure. In contrast to the non-metal-tolerant species, *G. claussenii* was able to store extremely high concentrations of these elements in shoots, eventually reaching 9.3 g Zn kg^-^^1^ dry weight (**Figure 5A**) and 1.3 g Cd kg^-^^1^ dry weight at the highest exposure levels (**Figure 5B**). The root:shoot accumulation ratios for Zn and Cd in *G. claussenii* averaged around two (**Figure 5**). *G. elegans* was clearly not tolerant to Cd, not even at the modest exposure level of 5 μM. Although the plants managed to keep Cd out of the shoot, the root concentrations at this exposure level already exceeded those found in *G. claussenii* at 10 μM Cd exposure (**Figure 6D**). At this concentration, more than 90% of the Cd was found in the roots of *G. elegans*. Although the shoot Cd concentrations were high, they remained significantly lower than those in *G. claussenii* shoots at 10 μM Cd exposure (*P* < 0.01; *n* = 3) (**Figure 6C**). At exposure to 100 μM Zn, the Zn concentrations in *G. elegans* roots and shoots were about 2, respectively 2.5 times lower than in *G. claussenii* (**Figures 6A,B**).

**FIGURE 4:**
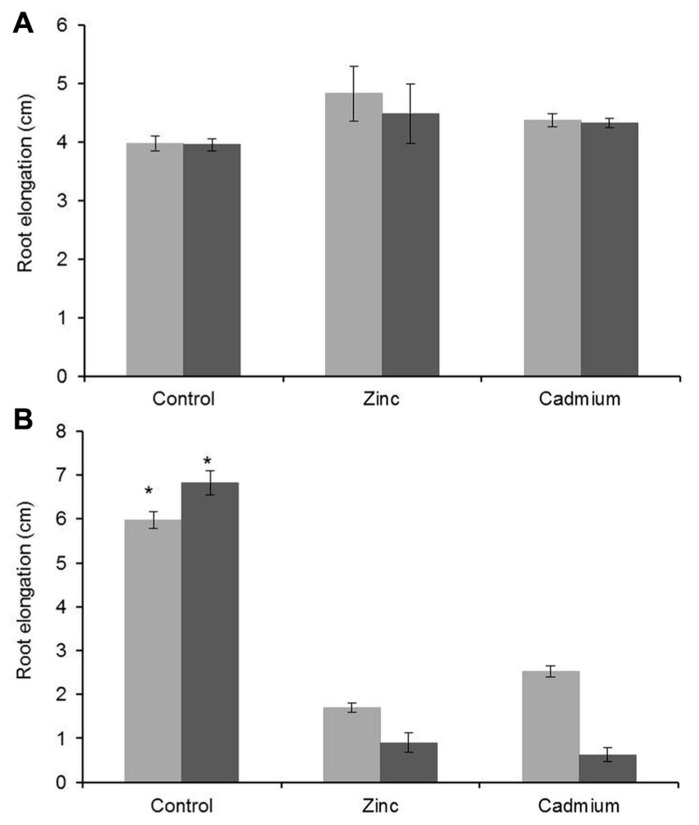
**Increase in root length of *G. claussenii* plants after 3 days (light gray bars) and 6 days (dark gray bars) of exposure to 2 μM zinc (Zn; control), 3000 μM Zn (zinc), and 100 μM Cd (cadmium) (A); and of *G. elegans* plants after exposure to 2 μM Zn (control), 100 μM Zn (zinc), and 5 μM Cd (cadmium) (B).** Mean values and standard errors are shown (*n* = 6). Asterisks denote significant differences of control (2 μM Zn) from treatments as found upon Tukey’s testing (*P* < 0.05).

**FIGURE 5:**
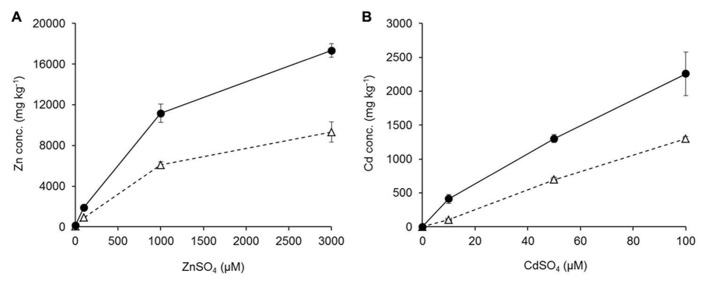
**Zinc (Zn) (A) and cadmium (Cd) concentrations (B) (in mg kg^–1^ dry weight; mean ± SE) of *G. claussenii* shoots (Δ) and roots (•) upon growth in hydroponic nutrient solutions.** Plants were grown for 3 weeks in a hydroponic solution containing 2 μM ZnSO_4_ before exposure to elevated ZnSO_4_ (100, 1000, and 3000 μM) or CdSO_4_ concentrations (0, 10, 50, and 100 μM).

**FIGURE 6:**
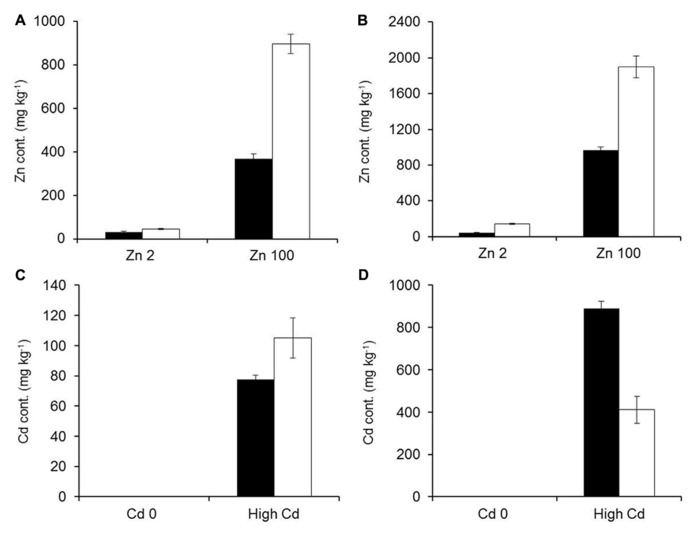
**Comparison between *G. elegans* (black bars) and *G. claussenii* (white bars) zinc (Zn) and cadmium (Cd) concentrations (mg kg–1; mean ± SE) in shoots (A,C) and roots (B,D) of plants exposed to comparable Zn (A,B) or Cd treatment levels (C,D).** Zn treatment concentrations were 2 and 100 μM of ZnSO_4_ (Zn2, Zn100). Cd treatment concentrations were either no exposure (Cd0) or 5 and 10 μM of CdSO_4_ to *G. elegans* and *G. claussenii*, respectively (high Cd). Plants were grown for 3 weeks on control solution (2 μM Zn) before 3 weeks of treatments exposure.

**FIGURE 7:**
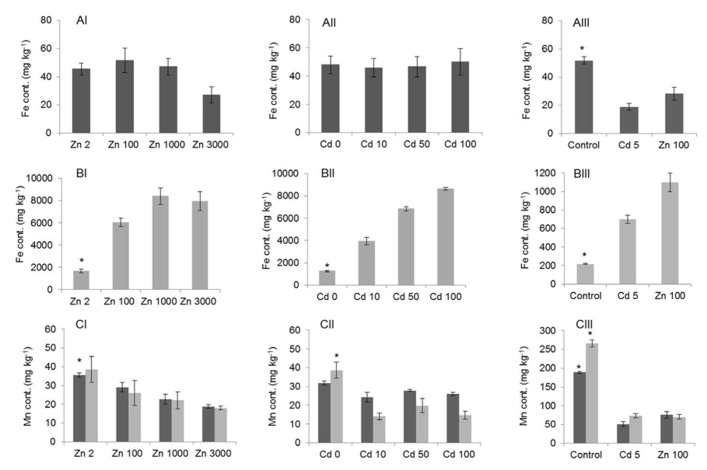
**Iron (Fe) (A,B) and manganese (Mn) (C) concentrations (mg kg^–1^; mean ± SE) in *G. claussenii* (I,II) and *G. elegans* (III) shoot (dark gray bars) and root (light gray bars).** Plants were grown for 3 weeks on control solution (Zn 2 μM) before exposure to Zn (2, 100, 1000, and 3000 μM) and Cd (0, 10, 50, and 100 μM) treatments for *G. claussenii* and to Zn (2 and 100 μM) and Cd (5 μM) treatments for *G. elegans*. Asterisks denote significant differences of control (Zn 2 μM) from treatments by Tukey’s test (*P* < 0.05).

The exposure to elevated Zn and Cd concentrations was also expected to affect the concentrations of other minerals, such as Fe and manganese (Mn), for which homeostasis mechanisms often interact with those for Zn. *G. claussenii* and *G. elegans* Fe concentrations in roots increased with an increase of Zn and Cd supply (**Figures 7BI,BII,BIII**). Fe concentrations in *G. claussenii* shoots were statistically similar (*P* > 0.05; *n* = 3) at all Zn and Cd exposure levels, even though at the highest Zn exposure, the Fe concentration appeared to be lower (**Figures 7AI,AII**). In *G. elegans*, the shoot Fe concentrations decreased significantly (*P* < 0.01; *n* = 3) at both Zn and Cd treatments (**Figure 7AIII**). Mn concentrations were also affected by the Zn and Cd treatments (**Figure 7C**). Although the Mn concentration in roots of Zn-exposed *G. claussenii* plants seemed to decrease at the highest exposure level, this was statistically not significant (*P* > 0.05; *n* = 3). The Mn concentration in shoots decreased significantly (*P* < 0.05) with increasing Zn exposure levels (**Figure 7CI**). When exposed to Cd, Mn decreased drastically in roots but stayed similar in shoots (**Figure 7CII**). There was no difference between metals treatment in *G. elegans*, both Zn and Cd treatments significantly reduced the Mn concentrations in roots and shoots (*P* < 0.01; *n* = 3; **Figure 7CIII**).

## DISCUSSION

The results we present here demonstrate that *G. claussenii* is indeed a novel metallophyte, extremely tolerant to high Zn and Cd exposure. This is the first report of a species of this kind from South America. Field results, together with pot experiments using soil from contaminated sites, present clues about the high Zn and Cd tolerance. These results also give an indication of the potential of this species for phytoremediation purposes under field-like conditions (Chaney et al., 2007), where mixed contaminations are more rule than exception (Kabata-Pendias and Mukherjee, 2007b). While the high metal tolerance is not unexpected, given the abundance of the species at the Zn mining site, the high levels of accumulation are surprising. The reported shoot Zn and Cd concentrations of *G. claussenii* plants grown on contaminated soil collected at the site (maximum 5300 and 280 μg g^-^^1^ for Zn and Cd, respectively; **Table 1**) clearly exceed the recently proposed threshold levels to classify species as metal hyperaccumulators, which are 3000 μg Zn g^-^^1^ dry weight and 100 μg Cd g^-^^1^ dry weight (van der Ent et al., 2012). This means *G. claussenii* is not only a metallophyte, but also a Zn/Cd hyperaccumulator species, again the first one known from the South American continent.

The hydroponic metal exposure experiments we performed subsequently provided an excellent way to evaluate the maximum levels of Zn/Cd tolerance and accumulation. A crucial point to consider when using extremely high levels of metals in hydroponic solutions is the metal availability (Baker and Whiting, 2002). We preferred to use half-strength Clark’s nutrient solution (Clark, 1975) for hydroponics, which is different from the more often used half Hoagland’s solution (Hoagland and Arnon, 1940), mainly because it allowed us to expose plants to higher Zn concentrations without precipitation of metals (**Table 2**).

To evaluate metal tolerance and accumulation of *G. claussenii*, we used the closely related species *G. elegans* as comparison, which is common in many South American countries (Mussury et al., 2006) and not known to be adapted to heavy metal exposure. Although both species are taxonomically close, they are clearly separate species, with different plant morphologies. Also our attempts to cross both species have not been successful. For the evaluation, we considered the effect of metal exposure on both roots and shoots, which was possible when using hydroponic conditions. Upon metal exposure, root growth is more rapidly affected than that of other plants parts, therefore root elongation has previously been suggested to be an efficient parameter to evaluate metal tolerance (Macnair et al., 1993). The treatment effects on root elongation easily distinguished both species in the highly Zn and Cd tolerant *G. claussenii* and the non-tolerant *G. elegans* (**Figure 4**).

*Gomphrena claussenii* not only adapted to high metal exposure by evolving metal tolerance traits, but it also evolved the capacity to store substantial amounts of Zn and Cd within the plant. The concentrations of Zn and Cd accumulated in root and shoot tissues (**Figure 5**) were approximately 10 times higher than what is reported to be toxic for most plant species (Kabata-Pendias and Mukherjee, 2007a). The differences in Zn accumulation between *G. claussenii* and *G. elegans*, when exposed to the same Zn level, were not as prominent as would be expected based on comparisons of other hypertolerant species with their closest non-tolerant relatives (Lasat et al., 1996; Ni et al., 2004). This may be a consequence of the natural high Zn concentration which is found in shoots of Amaranthaceae species. From 48 studied families, Amaranthaceae species have in average the second highest Zn concentration in shoots, 108 mg Zn kg^-^^1^ dry weight, while the average over all families was 77 mg Zn kg^-^^1^ dry weight (Broadley et al., 2007). Thus this plant lineage may be more prone to evolve Zn/Cd tolerance than other families.

Throughout the metal exposure treatments, *G. claussenii* showed hardly any signs of metal toxicity, not only in roots but also not in shoots, confirming its exceptional Zn and Cd tolerance. Only few other Zn and Cd hypertolerant species have been reported so far, such as *A. halleri* (Küpper et al., 2000), *Noccaea* (*Thlaspi*) *caerulescens* (Assunção et al., 2003a), *N. praecox* (Pongrac et al., 2009), *Sedum alfredii* (Yang et al., 2004), and *Viola baoshanensis* (Wu et al., 2010). However, different from these species that accumulate high levels of metals in shoots when exposed to low concentrations, *G. claussenii* presents an almost constant ratio between exposed and accumulated metal concentrations, typical of a metal bioindicator species (van der Ent et al., 2012). Thus, *G. claussenii* appears to have evolved another tolerance mechanism. The adaptive mechanism which evolved in classical hyperaccumulators is focused on preferentially accumulating metals in leaves to deter herbivores (Boyd, 2007; Fones et al., 2010). Instead, *G. claussenii* accumulates metals at approximately twice the concentrations in roots than in shoots (**Figure 4**), which indicates the ability to use shoots to store metals if storage capacity in roots is not adequate. This adaptation would probably require less modifications to the metal homeostasis mechanism than the evolution of metal hyperaccumulation. Releasing the barrier to prevent Zn and Cd translocation to the shoots, allowing the metals to follow the concentration gradient, would be sufficient. This is likely to involve genes of the heteroduplex mobility assay (HMA)-like P-type ATPase metal transporters, which are involved in loading metals into the xylem (Wong and Cobbett, 2009). Of course an increased metal flux from roots to shoots should be dealt with by providing sufficient apoplastic and vacuolar metal storage capacity in shoots, otherwise plants will accumulate metals in shoots, but not tolerate them and succumb to the toxic consequences. The mechanism for this can be similar for root or shoot tissues, and does not require the tight tissue-specific regulation of metal transporter gene expressions as found in hyperaccumulators, where roots appear to be actively involved in transporting metals to the vascular system and up into the shoots to keep root concentrations relatively low and shoot levels high, against the concentration gradient (Verbruggen et al., 2009). Such could simply be achieved by increasing expression of transporters exporting metals from the cytoplasm, either to the apoplast or to the vacuoles.

The effects of Zn or Cd exposure on Fe and Mn homeostasis were clearly higher in *G. elegans* than in *G. claussenii*. The decrease of Fe concentration in *G. elegans* shoots is a common effect of Zn and Cd toxicity in metal sensitive plants. In contrast, tolerant species like *G. claussenii* are able to keep shoot Fe concentrations unaffected (Shanmugam et al., 2011), avoiding the drastic symptoms that disturbance of Fe homeostasis will cause on photosynthesis. Root Fe concentrations in both species increased with increasing Zn or Cd exposure. For *G. elegans*, the increase of Fe in roots can be explained as a consequence of Fe deficiency in shoots, due to competition for uptake of Fe with Zn or Cd. The effect is much more pronounced for *G. claussenii* than for *G. elegans*. Metal hyperaccumulator species like *S. alfredii* and *N. caerulescens* also show an increase of root Fe concentration in response to high Zn or Cd exposure (Zhou and Qiu, 2005; van de Mortel et al., 2006). However, even though the solution speciation analysis showed that more than 90% of the Zn and Cd are available as free ions, the possibility that the high Fe concentration in roots of *G. claussenii* is a consequence of apoplastic Fe precipitation, rather than symplastic uptake, cannot be discarded (Chaney et al., 2007).

The combination of high metal tolerance and high metal accumulation along with high biomass production makes plants suitable for phytoextraction. Two major strategies have been considered to achieve these properties: to breed or genetically engineer hyperaccumulator species to increase their biomass or to genetically engineer high-biomass species to increase their metal accumulation and tolerance capacity (Chaney et al., 2007). These are not trivial challenges, but the main reason for this is the scarcity of natural metal hypertolerant and metal accumulating species that are high biomass producing. *G. claussenii* is a Zn/Cd accumulating species, which produces considerable biomass in the field, and we believe that domestication of this species can be a promising approach to consider for non-GMO-based phytoextraction.

## Conflict of Interest Statement

The authors declare that the research was conducted in the absence of any commercial or financial relationships that could be construed as a potential conflict of interest.
