# Erratum on: *Gomphrena claussenii*, the first South American metallophyte species with indicator-like Zn and Cd accumulation and extreme metal tolerance

**DOI:** 10.3389/fpls.2014.00509

**Published:** 2014-10-06

**Authors:** Frontiers Production Office

**Affiliations:** Frontiers Production Office, FrontiersSwitzerland

**Keywords:** phytoremediation, Zn/Cd hypertolerance, hyperaccumulation, metal contamination, *Gomphrena claussenii*, *Gomphrena elegans*

Reason for Erratum:

The citation for the author Mina Tomaz Villafort Carvalho was listed as Carvalho, MTV instead of Villafort Carvalho, MT, due to a typesetting error. This mistake does not change the scientific conclusions of the article in any way. The publisher apologizes for this error.

The original article has been updated.

